# Expression of the chemokine CXCL14 and cetuximab-dependent tumour suppression in head and neck squamous cell carcinoma

**DOI:** 10.1038/oncsis.2016.43

**Published:** 2016-07-11

**Authors:** T Kondo, S Ozawa, T Ikoma, X-Y Yang, K Kanamori, K Suzuki, H Iwabuchi, Y Maehata, C Miyamoto, T Taguchi, T Kiyono, E Kubota, R-I Hata

**Affiliations:** 1 Department of Reconstructive Oral and Maxillofacial Function, Graduate School of Kanagawa Dental University, Yokosuka, Japan; 2 Oral Health Science Research Center, Graduate School of Kanagawa Dental University, Yokosuka, Japan; 3 Department of Oral Science, Graduate School of Kanagawa Dental University, Yokosuka, Japan; 4 Department of Biology and Function in the Head and Neck, Yokohama City University School of Medicine, Yokohama, Japan; 5 Division of Carcinogenesis and Cancer Prevention, National Cancer Center Research Institute, Tokyo, Japan

## Abstract

Cetuximab, a monoclonal antibody against the epidermal growth factor receptor (EGFR), has been successfully used to treat some patients with colorectal cancer and those with head and neck squamous cell carcinoma (HNSCC). For the effective treatment, it is essential to first identify cetuximab-responsive patients. The level of EGFR expression and/or the presence of mutations in signalling molecules downstream of the EGFR pathway have been reported to be determining factors for cetuximab responsiveness in colorectal cancer patients; however, limited data have been reported for HNSCC patients. We previously reported that the chemokine CXCL14 exhibits tumour-suppressive effects against xenografted HNSCC cells, which may be classified into two groups, CXCL14-expressing and non-expressing cells under serum-starved culture conditions. Here we employed CXCL14-expressing HSC-3 cells and CXCL14-non-expressing YCU-H891 cells as representatives of the two groups and compared their responses to cetuximab and their CXCL14 expression under various conditions. The growth of xenografted tumours initiated by HSC-3 cells, which expressed CXCL14 *in vivo* and *in vitro*, was suppressed by the injection of cetuximab into tumour-bearing mice; however, neither the expression of the chemokine nor the cetuximab-dependent suppression of xenograft tumour growth was observed for YCU-H891 cells. Both types of cells expressed EGFR and neither type harboured mutations in signalling molecules downstream of EGFR that have been reported in cetuximab-resistant colon cancer patients. The inhibition of the extracellular signal-regulated kinase (ERK) signalling increased the levels of *CXCL14* messenger RNA (mRNA) in HSC-3 cells, but not in YCU-H891 cells. We also observed that the *CXCL14* promoter region in YCU-H891 cells was hypermethylated, and that demethylation of the promoter by treatment with 5-aza-2′-deoxycytidine restored *CXCL14* mRNA expression and *in vivo* cetuximab-mediated tumour growth suppression. Finally, we observed *in vivo* tumour growth suppression when YCU-H891 cells were engineered to express *CXCL14* ectopically in the presence of doxycycline. These results indicate that *CXCL14* expression may be a good predictive biomarker for cetuximab-dependent tumour suppression.

## Introduction

Head and neck cancer is the sixth most common cancer worldwide. Globally ~650 000 new cases of head and neck squamous cell carcinoma (HNSCC) are diagnosed each year.^1^

The use of monoclonal antibodies for cancer therapy has achieved considerable success in recent years.^2, 3^ One such antibody is cetuximab, which is a human–mouse chimeric monoclonal IgG1 antibody targeted against the epidermal growth factor receptor (EGFR).^1, 4, 5^ Recently, cetuximab has been used to treat patients with colorectal cancer and HNSCC. Cetuximab exhibits tumour-suppressive effects in some patients through EGFR signal blockade and antibody-dependent cellular cytotoxicity.^6, 7^ When cetuximab was used to treat HNSCC patients in conjunction with radiation therapy and anticancer agents such as cisplatin, patient survival was successfully prolonged.^8, 9, 10, 11^ The following factors are known to influence the tumour-suppressive effects of cetuximab: the expression level of EGFR in the tumour cells^12, 13, 14^ and the presence of mutations in *KRAS* (codons 12, 13, 61 and 146),^15, 16, 17^ *BRAF* (codon 600)^17^ and *PIK3CA* (codons 542, 545 and 1047).^18, 19, 20^ KRAS, BRAF or PIK3CA are signalling molecules acting downstream of EGFR. However, even in the absence of mutations in the above-mentioned genes, cetuximab does not exhibit tumour-suppressive effects in many patients. Thus, it is essential to discover a new method for identifying cetuximab-responsive patients.

In addition to gene mutations, abnormal gene expression in cancer cells may be caused by epigenetic modifications, including DNA methylation, histone modifications and changes in chromatin structure, all of which play crucial roles in a wide variety of biological processes, including the growth and differentiation of normal cells.^21, 22, 23, 24^ Currently, a new chemotherapeutic approach using 5-aza-2′-deoxycytidine (DAC), which focuses on reversing DNA hypermethylation, is being successfully employed to treat myelodysplastic syndrome.^25, 26^

Chemokines (chemotactic cytokines) belong to a group of structurally related proteins with molecular sizes in the range of 8–12 kDa, and they have been reported to regulate cellular trafficking in various types of cells. The non-ELR-motif chemokine CXCL14,^27^ which lacks a Glu–Leu–Arg tripeptide sequence adjacent to the CXC motif, is a homoeostatic chemokine that reportedly stimulates the chemotaxis of B cells and monocytes,^28^ dendritic cells^29, 30^ and natural killer cells,^31, 32^ and also suppresses angiogenesis.^29, 33^ CXCL14 is known to function as a tumour suppressor in HNSCC,^34, 35^ breast cancer,^36^ lung cancer^37^ and hepatocellular carcinoma.^38^ In a previous study, we demonstrated that *CXCL14* expression is significantly downregulated by the activation of EGFR signalling,^34^ and that the restoration of *CXCL14* expression contributes to the tumour-suppressive effect of gefitinib, a selective tyrosine kinase inhibitor of EGFR.^39^ Recently, CXCL14 expression was demonstrated to be silenced by DNA hypermethylation in many malignant tumours, including lung cancer,^37^ colon cancer,^40^ stomach cancer^41^ and acute myeloid leukaemia.^42^ The promoter region of *CXCL14* contains CpG islands, and two GC boxes located in the −14 to −9 bp and −10 to −5 bp regions located upstream of the transcriptional start site; these GC boxes play important roles in the expression of the *CXCL14* gene.^43^

In this study, using methylation levels of the *CXCL14* promoter as a marker, we investigated whether DNA hypermethylation contributes to the tumour-suppressive effect of cetuximab. Additionally, we investigated the use of DAC in HNSCC cells for the demethylation of DNA. We demonstrated that DAC increased the expression of *CXCL14* messenger RNA (mRNA) and enhanced the tumour-suppressive effect of cetuximab.

## Results and discussion

Previously, we subcutaneously injected four HNSCC cell lines into athymic nude mice and treated the mice with intraperitoneal injections of gefitinib (ZD1839, trade name Iressa, AstraZeneca, Osaka, Japan), a selective inhibitor of the tyrosine kinase of EGFR.^39^

Tumour growth was significantly suppressed in three groups of mice injected with HSC-2 (oral floor carcinoma-derived), HSC-3 (tongue carcinoma-derived) or HSC-4 (tongue carcinoma-derived) cells, concomitant with an increase in *CXCL14* mRNA expression. However, tumour growth in mice injected with YCU-H891 (hypopharynx carcinoma-derived) cells was not suppressed, nor was CXCL14 expression observed,^39^ suggesting that CXCL14 expression may be a marker for the suppression of tumour growth. To investigate whether CXCL14 expression is a marker of tumour suppression mediated by cetuximab, we used six HNSCC cell lines.

**Figure 1:**
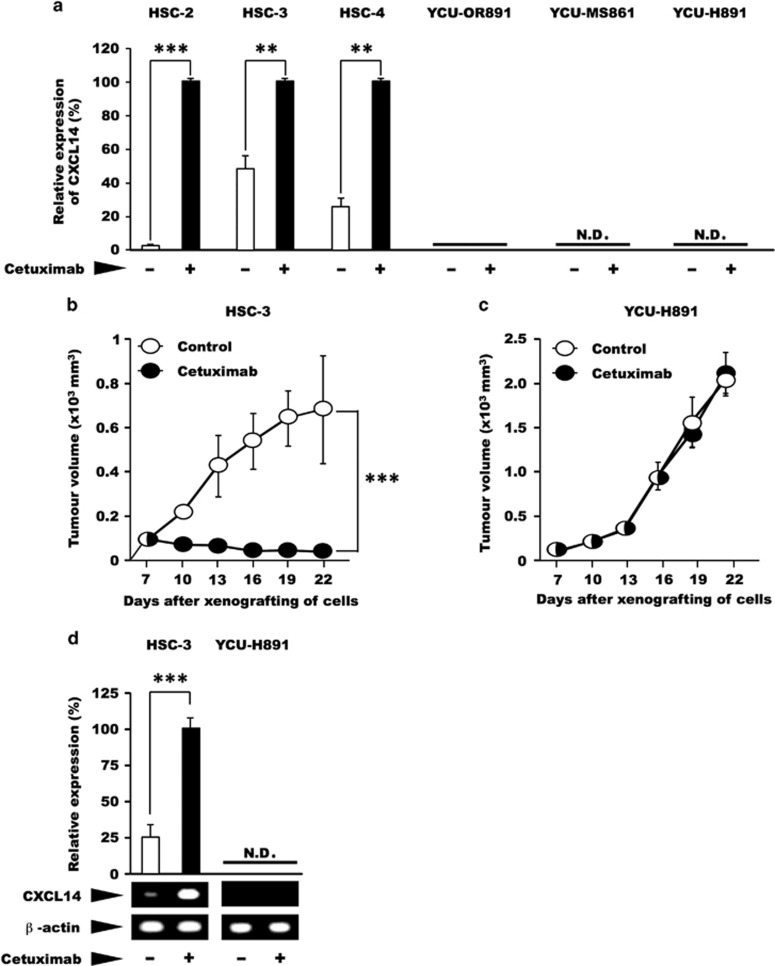
Effects of cetuximab on the expression level of *CXCL14* mRNA and tumour volume. (**a**) In an *in vitro* experiment, oral floor carcinoma-derived HSC-2 cells, tongue carcinoma-derived HSC-3 and HSC-4 cells, obtained from the Japanese Collection of Research Bioresources Cell Bank, as well as oral floor carcinoma-derived YCU-OR891 cells, maxillary sinus carcinoma-derived YCU-MS861 cells and hypopharynx carcinoma-derived YCU-H891 cells, which were established in our laboratory in a previous study,^39^ were cultured in Dulbecco's Modified Eagle's medium (DMEM) (Sigma-Aldrich, St Louis, MO, USA) containing 50 μg/ml gentamicin sulphate (Wako Pure Chemical Industry, Osaka, Japan) and 10% fetal bovine serum (Wako or Thermo Fisher Scientific, Yokohama, Japan) at 37 °C under 95% air and 5% CO_2_ until the cells reached the pre-confluent stage. The cells (1 × 10^5^ per well) were then inoculated into three wells of six-well plates (Corning, Tokyo, Japan). After 2 days in culture, the cells were treated with cetuximab (10 μg/ml Bristol-Myers Squibb Company, New York City, NY, USA) or control for 24 h. Total RNA was subsequently extracted and purified using TRIzol (Thermo Fisher Scientific). Total RNA (1 μg) was reverse transcribed to complementary DNA (cDNA) using a PrimeScript RT Reagent kit with gDNA Eraser (Perfect Real Time: Takara, Kusatsu, Japan). Reactions for reverse transcription proceeded according to the manufacturer's instructions: 42 °C (gDNA removal) for 2 min, 37 °C (reverse transcription) for 15 min and 85 °C (inactivation of the reverse transcriptase) for 5 s. All quantitative PCR (qPCR) experiments were performed using an Applied Biosystems StepOnePlus Real-Time PCR Systems (Applied Biosystems, Tokyo, Japan). All amplifications were performed with Power SYBR Green PCR Master Mix (Life Technologies, Warrington, UK). Primer sets were as follows: for human CXCL14, 5′-AAGCCAAAGTACCCGCACTG-3′ (forward) and 5′-GACCTCGGTACCTGGACACG-3′ (reverse), which yielded a 73-bp product; and for human β-actin, 5′-GTGAAGGTGACAGCAGTCGGTT-3′ (forward) and 5′-GAAGTGGGGTGGCTTTTAGGAT-3′ (reverse), which yielded a 157-bp product. The thermal cycling conditions included an initial denaturation step at 95 °C for 10 min, followed by 40 cycles at 95 °C for 15 s and 60 °C for 1 min. Melting curve analysis of every qPCR was conducted after each cycle. Specific amplification was confirmed by checking the melting curves and melting temperatures of the qPCR products. Experiments were performed in triplicate, and the values were normalized to β-actin. (**b**, **c**) For the *in vivo* experiments, we subcutaneously inoculated BALB/c nude mice (female, 5-week old, Clea Japan Inc., Tokyo, Japan) on the dorsal side with 1 × 10^7^ HSC-3 or YCU-H891 cells (12 mice per group). Seven days after cell inoculation (at a tumour size of ~100 mm^3^), we intraperitoneally administered cetuximab (10 mg/kg) or Dulbecco's phosphate-buffered saline (DPBS, Wako) at random to the animals three times per week and measured the tumour size once every 3 days for the HSC-3 cells (**b**) and YCU-H891 cells (**c**). Tumour volumes were measured by a person different from the one who injected cetuximab or DPBS once every 3 days and were calculated using the formula, (a × b × b)/2, where ‘a' is the long diameter and ‘b' is its short diameter of the tumours. (**d**) To determine the expression levels of *CXCL14* mRNA *in vivo*, we removed the tumours 22 days after inoculation, isolated the total RNA and measured the expression level of *CXCL14* mRNA in the HSC-3 and YCU-H891 tumours. *CXCL14* cDNA was synthesized by performing the reverse transcription–PCR (RT–PCR) with SuperScript II reverse transcriptase (Invitrogen, Thermo Fisher Scientific K.K. Yokohama, Japan) and Ex Taq DNA polymerase (Takara, Otsu, Japan). Brilliant SYBR Green qPCR Master Mix was obtained from Stratagene (La Jolla, CA, USA). The following primers were used for RT–PCR: primers for human *CXCL14*, 5′-AAT GAA GCC AAA GTA CCC GC-3′ (forward) and 5′-AGT CCT TTG CAC AAG TCT CC-3′ (reverse; PCR product size, 230 bp); and primers for β-actin, 5′-AAA GAC CTG TAC GCC AAC AC-3′ (forward) and 5′-CTC GTC ATA CTC CTG CTT GC-3′ (reverse; PCR product size, 222 bp). The PCR cycling conditions included denaturation at 94 °C for 30 s, annealing at 58 °C for 30 s and elongation at 72 °C for 30 s. The PCR products were separated on 2% agarose gel by electrophoresis and were visualized with ethidium bromide dye.^45^ β-Actin cDNA was used as an internal standard and for normalisation. qPCR and/or densitometry were employed for quantitative comparison of the expression levels of *CXCL14* mRNA between the two groups. These experiments were repeated twice. The *in vivo* experiments were performed in accordance with the local guidelines for the welfare of experimental animals and with the approval of the Ethics Committee on Animal Research of Kanagawa Dental University. The animals were housed in temperature-controlled rooms and received water and food *ad libitum*. We followed the guidelines for animal research of the International Association for the Study of Pain Committee for Research and Ethical Issues. In these experiments, Student's *t-*test was used to evaluate statistically significant differences between any two groups. N.D.; not detected. The values are expressed as the mean±s.d. (*n*=6). Half-white, half-black circles represent overlapping points. A *P*-value<0.05 was considered statistically significant. ***P<0.001 and **P<0.01. For some of the data points, the s.d. values were smaller than the size of the symbols used.

In an *in vitro* culture system, treatment with cetuximab led to a 2- to 50-fold increase in the expression of *CXCL14* mRNA in HSC-2, HSC-3 and HSC-4 cells (*P*<0.01; Figure 1a, left panel). Conversely, in YCU-MS861 (maxillary sinus carcinoma-derived) and YCU-H891 cells, no expression of *CXCL14* mRNA was detected, in the presence or absence of *in vitro* cetuximab (Figure 1a, right panel). Interestingly, YCU-OR891 (oral floor carcinoma-derived) cells expressed a very small amount of CXCL14, but the level was not stimulated by the treatment with cetuximab (Figure 1a). Next, we investigated the *in vivo* tumour-suppressive effect of cetuximab on the HSC-3 and YCU-H891 cell lines, as representatives of the two groups, by dorsal inoculation of nude mice with these tumour cells. Cetuximab exerted significant tumour-suppressive effect against xenografted HSC-3 cells (*P*<0.001; Figure 1b). By contrast, this monoclonal antibody was ineffective against the xenografted YCU-H891 cells (Figure 1c). The administration of cetuximab caused an approximately fourfold increase in the expression of *CXCL14* mRNA levels in the HSC-3 tumours (*P*<0.001; Figure 1d, left panel). Conversely, in the YCU-H891 tumours, no expression of *CXCL14* mRNA was detected upon the administration of cetuximab (Figure 1d, right panel). These results indicate that the treatment with cetuximab *in vitro* and in *vivo*, stimulated the expression of *CXCL14* mRNA in HSC-3 cells, but not in YCU-H891 cells. Furthermore, the expression of *CXCL14* mRNA in HSC-3 cells was associated with the suppression of tumour growth.

**Figure 2:**
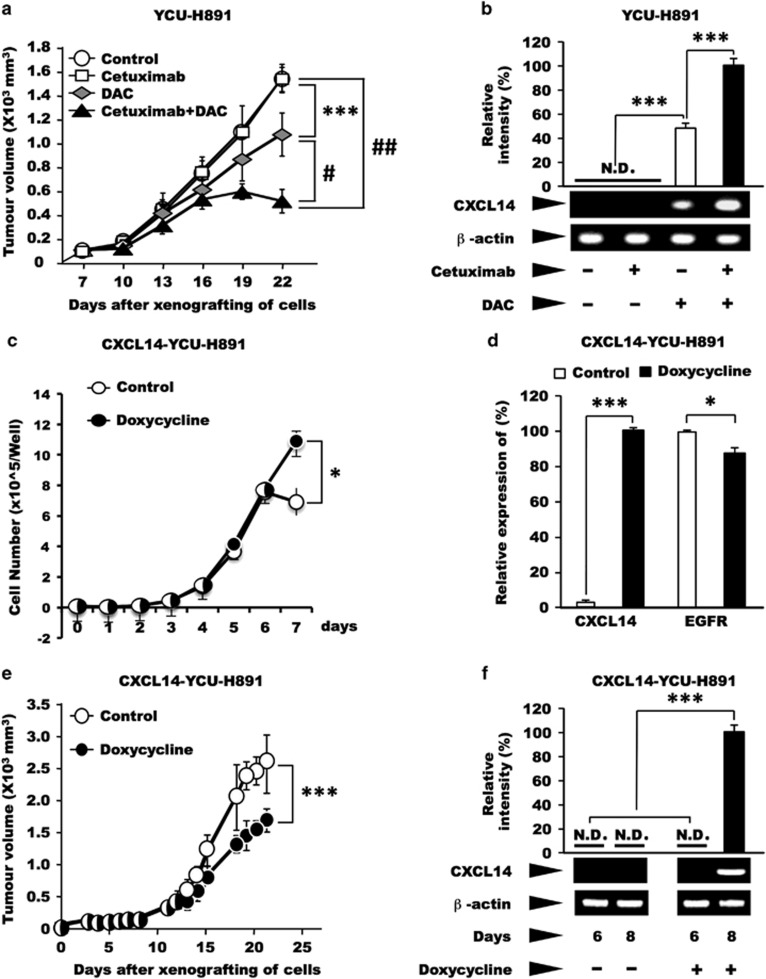

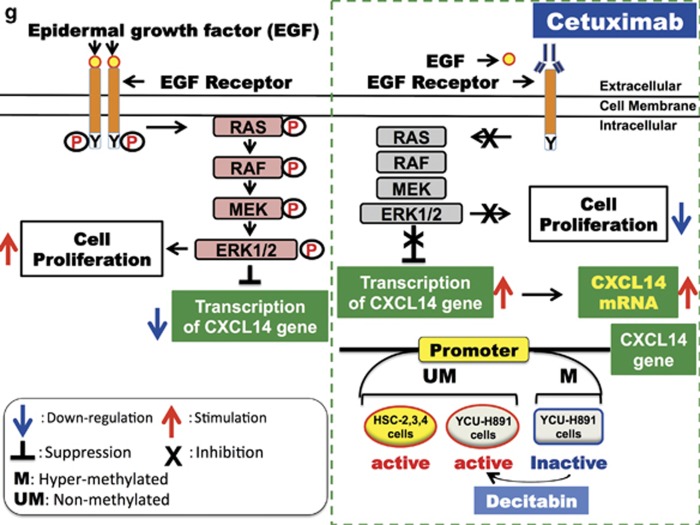
Sequence analysis of signalling molecules downstream of the epidermal growth factor receptor (EGFR) and the effects of inhibitors of PI3K, MEK and ERK MAPK kinases on *CXCL14* expression. (**a**) After DNA extraction, the nucleotide sequences commonly mutated in cetuximab-resistant colon cancer patients were analysed with a Gene JET Genomic DNA Purification kit (Thermo Fisher Scientific). After PCR amplification of the target sequences, the products were purified with the QIAquick Gel Extraction kit (Qiagen, Tokyo, Japan). We used the following primers for the PCR analyses: 5′-ACA CGT CTG CAG TCA ACT GG-3′ (forward) and 5′-GTC CTG CAC CAG TAA TAT GC-3′ (reverse; PCR product size, 338 bp) for *KRAS* codons 12 and 13; 5′-CTT TTC AGG TGC TTA GTG TC-3′ (forward) and 5′-AGC AAG TTA CTC CAC TGC TC-3′ (reverse; PCR product size, 538 bp) for *KRAS* codon 61; 5′-TTT TAT GAC AAA AGT TGT GGA CAG G-3′ (forward) and 5′-CCA AAG CCA AAA GCA GTA CC-3′ (reverse; PCR product size, 431 bp) for *KRAS* codon 146; 5′-GAA AGC ATC TCA CCT CAT CC-3′ (forward) and 5′-TAA TGG CTG TGG ATC ACA CC-3′ (reverse; PCR product size, 823 bp) for *BRAF* codon 600; 5′-GCT TTT TCT GTA AAT CAT CTG TGA ATC C-3′ (forward) and 5′-TGC AGA AAT GCA CTG CAA CTG G-3′ (reverse; PCR product size, 672 bp) for *PIK3CA* codons 542 and 545; and 5′-GCT TTG TCT ACG AAA GCC TC-3′ (forward) and 5′-GCT ATC AAA CCC TGT TTG CG-3′ (reverse; PCR product size, 560 bp) for *PIK3CA* codon 1047. The PCR cycling conditions were as follows: denaturation at 94 °C for 30 s, annealing at 58 °C for 30 s and elongation at 72 °C for 30 s. We used each of the forward and reverse primers to perform the sequencing reactions. We performed the analysis using a dye terminator cycle sequencing kit (Beckman Coulter, Tokyo, Japan) and a CEQ2000 sequencer (Beckman Coulter). The results of direct nucleotide sequencing of the following nucleotides are presented: *KRAS* codons 12, 13, 61 and 146; *BRAF* codon 600; and *PIK3CA* codons 542, 545 and 1047. These regions were not mutated in the HNSCC HSC-3 or YCU-H891 cells. (**b**–**f**) To investigate the effects of inhibitors of signalling molecules downstream of the EGFR on *CXCL14* expression, we cultured HSC-3 and YCU-H891 cells to the pre-confluent state as described in the Figure 1d legend, and treated with the PI3K inhibitor AS605240 (Echelon Biosciences, Salt Lake City, UT, USA, 10 μM; **b**) MEK inhibitor PD98059 (Merck Millipore, Darmstadt, Germany, 50 μM; **c**), MEK1/2 inhibitor U0126 (Merck Millipore, 10 μM; **d**) or ERK1/2 inhibitor FR180204 (Merck Millipore, 10 μM, **e**). Total RNA was extracted with TRIzol after 24 h of culture, and the expression levels of *CXCL14* and β-actin were determined by RT–PCR. For the HSC-3 cells (**b**–**e**) and YCU-H891 cells (**f**), *β-actin* cDNA was used as an internal standard and for normalisation. For quantitative comparison of the expression levels of *CXCL14* mRNA, qPCR and/or densitometry after agarose gel electrophoresis was performed. The values are expressed as the means±s.d. (*n*=3). ***P<0.001 (Student's *t*-test). Experiments were performed in triplicate, and the values were normalized to β-actin.

It has been reported that the non-responsiveness to the cetuximab treatment in colon cancer patients is due to mutations in signalling molecules acting downstream of EGFR, including mutations in the *KRAS*, *RAF* (cf. Figure 4g) and *PIK3CA* genes. We therefore determined the DNA sequences of these genes in HSC-3 and YCU-H891 cells by direct sequencing. No reported mutations were detected in either cell line in *KRAS* codons 12, 13, 61 or 146, *BRAF* codon 600 or *PIK3CA* codons 542, 545 or 1047 (Figure 2a).

To investigate the effects of EGFR downstream signalling on the expression of the *CXCL14* gene, we treated HSC-3 cells with inhibitors of the various signalling molecules. AS605240, a PI3K inhibitor, did not alter the expression of *CXCL14* in HSC-3 cells (Figure 2b), suggesting that Akt does not regulate *CXCL14* expression. By contrast, treatment with PD98059 or U0126, inhibitors of MEK, a molecule downstream of KRAS and RAF (Figure 2c and d) or with FR180204, an ERK inhibitor (Figure 2e) significantly increased the mRNA levels of *CXCL14* in HSC-3 cells (*P*<0.001). However, these inhibitors did not have any effect on the expression of *CXCL14* in YCU-H891 cells (Figure 2f). These data indicate that the non-responsiveness of YCU-H891 cells to cetuximab was not due to mutations in any signalling molecules downstream of EGFR, and they suggest a defect in the transcriptional activation of the *CXCL14* gene in these cells.

**Figure 3:**
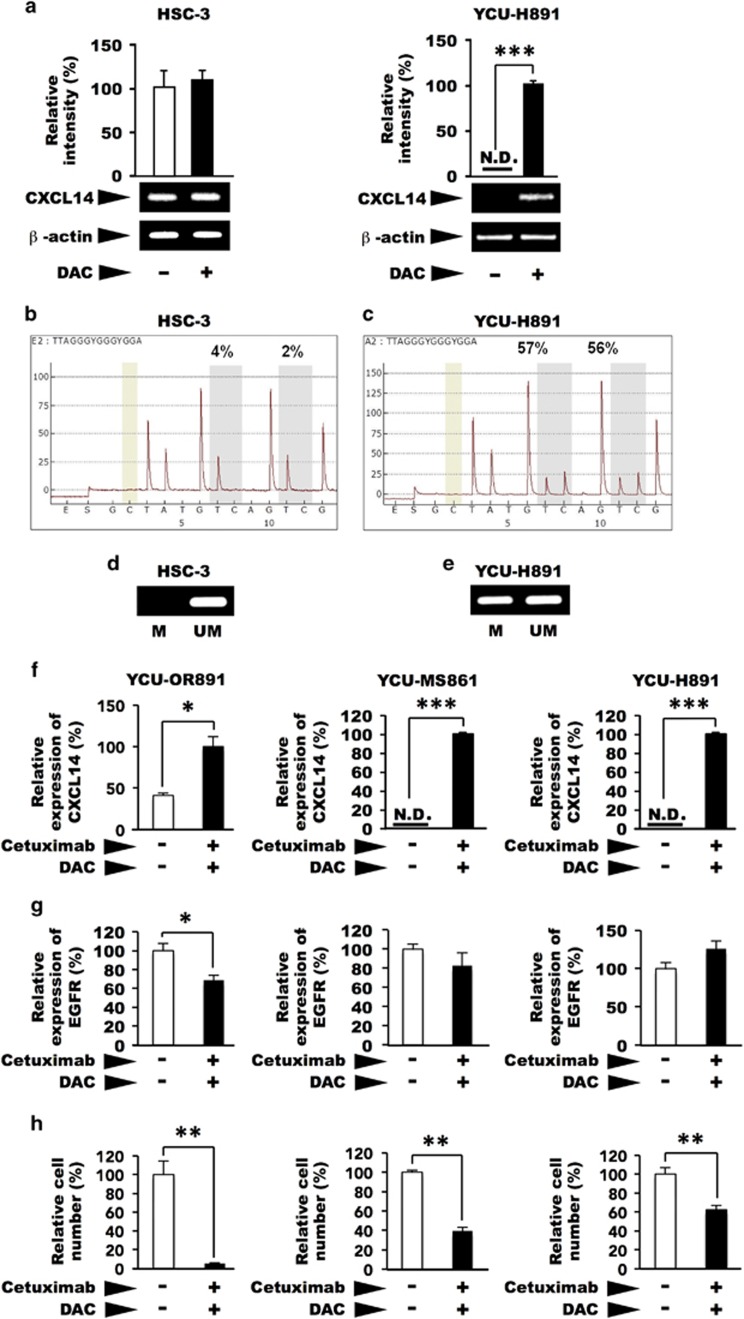
Effects of 5-aza-2′-deoxycytidine(DAC) on the expression of *CXCL14* mRNA and the methylation of the promoter region of *CXCL14* in HSC-3 and YCU-H891 cells. (**a**) Beginning 1 day after the cells had been plated (1.0 × 10^5^/60-mm dish, Corning) the medium was replaced every day with fresh medium containing DAC (Sigma-Aldrich, final concentration, 5 μM) or control. Total RNA was extracted after 3 days of culture, and the expression levels of *CXCL14* were determined by reverse transcription–PCR and quantitative PCR (qPCR) or densitometry after gel electrophoresis of the complementary DNA (cDNA), as described in the Figure 1d legend. Similar levels of *CXCL14* mRNA were observed in the treated and untreated control HSC-3 cells (left panel). However, *CXCL14* expression was detected only in the YCU-H891 cells only in the presence of DAC (right panel). (**b**, **c**) For the methylation analysis, we seeded 1 × 10^5^ HSC-3 and YCU-H891 cells per 60-mm dish and replaced the medium every day with fresh medium containing DAC (final concentration, 5 μM) starting on the following day. Three days after initiation of DAC treatment, we extracted RNA and verified the expression of *CXCL14* and β-actin. Using a QiaAmp DNeasy kit (Qiagen), we extracted the DNA, and the EZ DNA Methylation-Gold kit (Zymo Research, Irvine, CA, USA) was used to perform bisulphite substitution. Using the PSQ assay design program (Qiagen), we designed the following PCR primers for pyrosequencing: 5′-GYG GGT TGG GAA GGT TTT-3′ (forward primer), 5′-TCR ATA AAT ACC CAA AAC TAT CT-3′ (5′-biotinylated reverse primer; PCR product size, 206 bp) and 5′-ACG AG(C/T) GGA TTT AAA AGA GG-3′ (sequencing primer). The pyrosequencing analysis was performed with a PyroMark ID system (Qiagen) and a Pyro Gold Reagent kit RRK (Qiagen). The results for the HSC-3 cells (**b**), and YCU-H891 cells (**c**) are presented. For methylation-specific PCR, we used the MethPrimer program to design PCR primers. The cycling conditions for the methylation and non-methylation PCR reactions were as follows: denaturation at 95 °C for 5 min, 45 cycles of denaturation at 95 °C for 20 s, annealing at 60 °C for 30 s and elongation at 72 °C for 30 s, followed by additional elongation at 72 °C for 5 min. (**d**, **e**) We verified the PCR products by performing agarose gel electrophoresis (2% gel). The PCR products were visualized with ethidium bromide staining after gel electrophoresis (HSC-3 cells (**d**) and YCU-H891 cells (**e**)). The values in **a** are expressed as the means±s.d. (*n*=3). Methylated (M) and unmethylated (UM) primers were used. (**f**, **g**) YCU-MS861 cells and YCU-H891 cells were inoculated into 60-mm culture dishes and cultured as described in the description in **a**, except that the cells were treated with 1 μg of cetuximab per ml for the last 24 h. RNA was purified by TRIzol, and qPCR was performed as described in the legend for Figure 1d. The relative rates of cDNAs for human CXCL14 (**f**), and human EGFR (**g**) are presented. For human EGFR, 5′-TCCCCGTAATTATGTGGTGAC-3′ (forward) and 5′-GCCCTTCGCACTTCTTACAC-3′ (reverse) were employed to yield a 110-bp product. (**h**) For the determination of the growth properties of the cells, the cells were plated in 12-well plates (Sumitomo Bakelite, Tokyo, Japan, 5 × 10^4^ per well) and cultured as described above, except for the YCU-OR891 cells, which were inoculated at 1 × 10^4^ per well and cultured for 6 days. Cell numbers from three wells were counted using a Coulter Z1 Counter (Coulter Electronics Ltd, UK) and relative cell numbers are presented. The values are expressed as the means±s.d. (*n*=3). *P<0.05, **P<0.001 and ***P<0.001 (Student's *t*-test). Experiments were performed in triplicate, and the values were normalized to β-actin. The figures represent one of two cell-culture experiments.

Transcriptional activity is often suppressed by the methylation of cytidine residues in the promoter regions of genes. The *CXCL14* gene is often silenced because of hypermethylation in several types of malignant tumours.^37, 40, 41, 42^ Two GC boxes located upstream of the transcription start site are essential for the transcription of the *CXCL14* gene.^43^ Treatment of cultured HSC-3 cells with DAC, an inhibitor of cytidine methylation, did not affect *CXCL14* expression (Figure 3a, left panel). However, in YCU-H891 cells, *CXCL14* mRNA expression was significantly stimulated by DAC (Figure 3a, right panel), suggesting that the methylation of the two GC boxes upstream of the *CXCL14* transcription start site was responsible for the silencing of *CXCL14* in these cells. We then determined the methylation levels of both GC boxes pyrosequencing. In HSC-3 cells, the methylation levels were 4% at the first GC box and 2% at the second GC box (Figure 3b). However, in YCU-H891 cells, the methylation levels were 57% and 56% at the first and second GC boxes, respectively (Figure 3c). In addition, we used methylation-specific PCR to confirm methylation levels in HSC-3 and YCU-H891 cells. In HSC-3 cells, we detected amplified bands with the non-methylation primers (Figure 3d, UM), but not with the methylation primers (Figure 3d, M). By contrast, in the YCU-H891 cells, we detected bands with both the non-methylation (UM) and the methylation (M) primers (Figure 3e). These data indicate that hypermethylation upstream of the transcription start site was responsible for the transcriptional repression of the *CXCL14* gene in YCU-H891 cells. Next, we examined the effects of co-treatment with DAC and cetuximab on the cellular behaviour of YCU-OR891, YCU-MS861 and YCU-H891 cells, which did not increase the expression of CXCL14 in response to the treatment with cetuximab alone (Figure 1a, right panel). These cells expressed significant amounts of CXCL14 upon the co-treatment with cetuximab and DAC (Figure 3f and g), and the growth of these cells was significantly suppressed (Figure 3h). These cells also expressed the EGFR, but the expression levels were not stimulated by the co-treatment. By contrast, the growth of these cells was significantly suppressed by the co-treatment, suggesting that the growth rate of the cells was not merely a reflection of EGFR expression.

**Figure 4:**
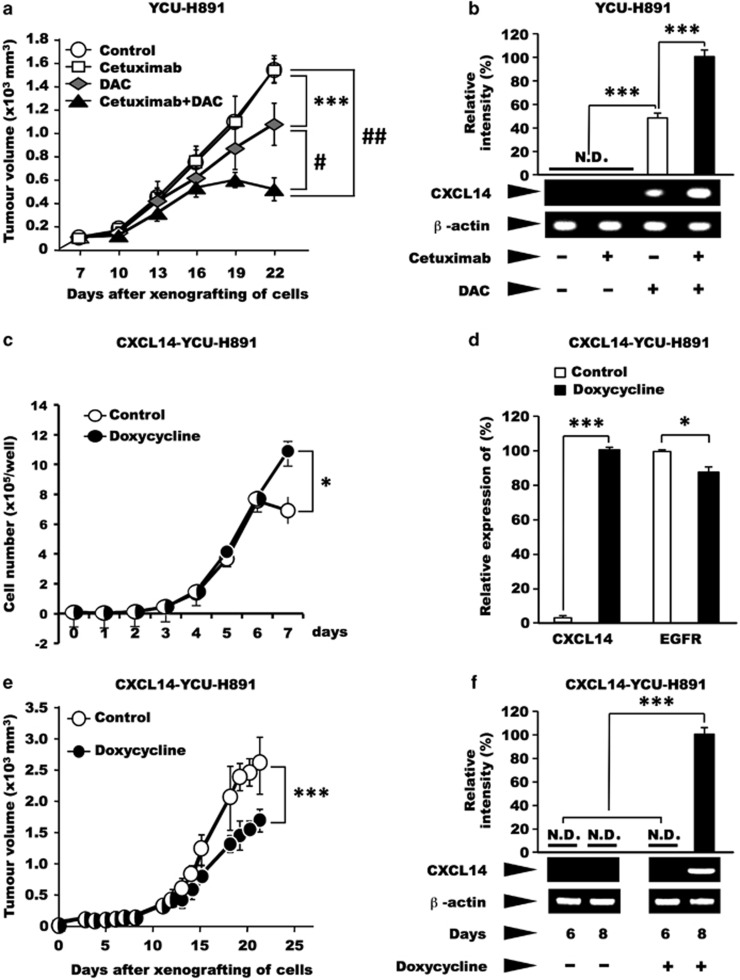

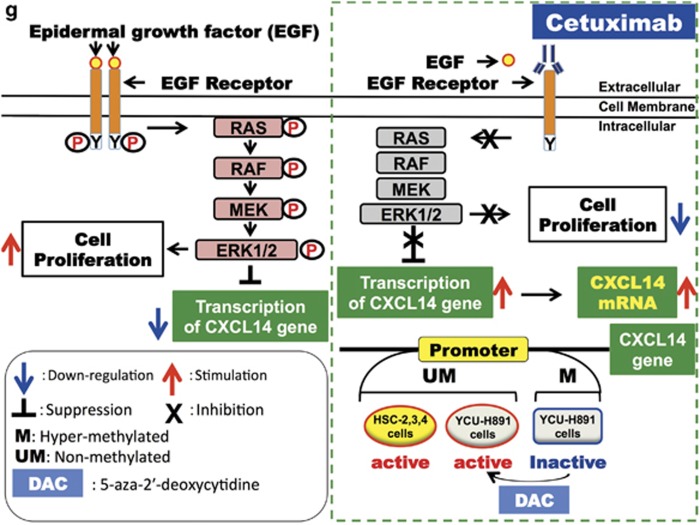
Effects of cetuximab and/or DAC, or ectopic expression of CXCL14 on tumour growth and *CXCL14* mRNA levels in YCU-H891 cells. (**a**, **b**) BALB/c nude mice (24 females, 5-week old) were subcutaneously inoculated on the dorsal side with YCU-H891 cells (1 × 10^7^ per site). Seven days after inoculation (at a tumour size of ~100 mm^3^), the animals were randomly divided into four groups and intraperitoneally administered Dulbecco's phosphate-buffered saline (control), cetuximab (cetuximab-only, 10 mg/kg), DAC (DAC-only, 5.0 mg/kg), or both cetuximab (10 mg/kg) and DAC (5.0 mg/kg; cetuximab+DAC) three times per week. Tumour volumes were measured once every 3 days by a person different from the one who injected the reagents (**a**) and were calculated with the formula, (a × b × b)/2, where ‘a' is the long diameter and ‘b' is its short diameter of the tumours. Total RNA was isolated, and the expression levels of *CXCL14* mRNA were determined as described in the legend for Figure 1d. To compare the expression levels of *CXCL14* between the DAC-only and cetuximab+DAC groups, we quantified the level of *CXCL14* mRNA in each group by using quantitative PCR (qPCR; **b**). One of the representative data set of two similar experiments is presented. (**c**, **d**) In another series of experiments, we engineered YCU-H891 cells to ectopically express *CXCL14* under the control of doxycycline (CXCL14-YCU-H891 cells). The cells (1 × 10^4^ per well) were plated into 24-well plates (Sumitomo Bakelite, Osaka, Japan) in DMEM medium containing 10% fetal bovine serum, and the next day half of the plates were treated with 0.2 μg/ml of doxycycline (Doxycycline, Takara). The experiments were repeated three times and yielded similar results. The number of cells was counted using a Coulter Z1 Counter (Coulter Electronics Ltd, London, UK; **c**). One day after the cells had been plated (5 × 10 × 10 × 10 × 10) into six-well plates, half of the plates were treated with 2 μg/ml doxycycline. After 24 h, RNA was isolated and qPCR was performed as described in the legend for Figure 3f and g, and relative expression levels of mRNAs of *CXCL14* and *EGFR* were determined (**d**). (**e**, **f**) For the *in vivo* experiment, the cells (1 × 10^7^ per site) were subcutaneously injected into the backs of BALB/c nude mice (12 female, 5-weeks old). Seven days after tumour cell inoculation (at a tumour size of ~100 mm^3^), the mice were randomly divided into two groups. One group was fed a 5% (w/v) sucrose solution containing 2 mg/ml doxycycline (Takara), whereas the other group was fed a 5% sucrose solution (control). The tumour sizes were measured five times per week, as described above (**e**). To confirm the enhanced expression of *CXCL14* in the presence and absence of doxycycline, we removed tumour tissue before (day 6) and after doxycycline administration (day 8), extracted the total RNA from the tissue samples and measured the levels of *CXCL14* mRNA (**f**), as described above. The values are expressed as the mean±s.d. (*n*=6). ***P<0.001, ^#^*P*<10^−4^, ^##^*P*<10^−5^ (Student's *t*-test). A schematic representation of the effects of EGF and cetuximab on cell proliferation and the expression of CXCL14 is presented in **g**.

Next, we investigated whether treatment of YCU-H891 tumours with a demethylating agent would restore the tumour-suppressive effect of cetuximab *in vivo*. The administration of cetuximab alone to animals bearing xenografted YCU-H891 cells resulted in tumour growth that was similar to that of the control group. However, tumour growth was significantly (*P*<0.001) suppressed in the DAC-only group (Figure 4a, *P*<10^−3^), compared with the control and cetuximab-only groups. Moreover, compared with the administration of DAC alone, the administration of cetuximab concurrently with DAC (cetuximab+DAC group), significantly suppressed the tumour growth-inducing ability of YCU-H891 (Figure 4a, *P*<10^−5^). Next, we extracted RNA from the tumours to measure their *CXCL14* expression. In the control and cetuximab-only groups, *CXCL14* expression was not detected (N.D. in Figure 4b); however, in the DAC-only and cetuximab+DAC groups, *CXCL14* expression was detected (Figure 4b). When we used quantitative PCR to compare *CXCL14* expression levels in the DAC-only and cetuximab+DAC groups, the results revealed that *CXCL14* expression was significantly higher in the cetuximab+DAC group than in the DAC-only group (*P*<0.001; Figure 4b).

Because the tumour suppression induced by DAC treatment may have been due to re-expression of tumour suppressor gene(s) other than *CXCL14*, we used a viral expression vector encoding *CXCL14* to express the gene in a doxycycline-dependent manner in YCU-H891 (CXCL14-YCU-H891) cells, which do not express endogenous *CXCL14*. When, we cultured CXCL14-YCU-H891 cells in the presence or absence of doxycycline, the presence of doxycycline did not suppress the increase in cell growth (Figure 4c), although doxycycline significantly stimulated the expression of *CXCL14* (Figure 4d). Under these conditions the expression of EGFR was not increased. These experiments were repeated three times and yielded reproducible results, suggesting that the expression of CXCL14 did not suppress the cell growth under the *in vitro* culture conditions employed. After 7 days, the cell number was higher in the presence of doxycycline, suggesting that the expression of CXCL14 may have affected the cell adhesion properties of the tumour cells, although this possibility requires further investigation.

To investigate the effect of CXCL14 expression in tumour cells on the growth of tumours *in vivo*, we subcutaneously inoculated nude mice with tumour cells on their dorsal side and orally administered doxycycline to the mice every day, beginning on day 7 post-tumour cell inoculation. The sizes of the tumours were significantly smaller in the doxycycline-administered group than the control group (*P*<0.001; Figure 4e). We extracted RNA from the tumours on post-inoculation day 6 (before the administration of doxycycline) and day 8 (after the administration of doxycycline), and examined the expression of *CXCL14* mRNA by quantitative PCR. On day 6, *CXCL14* expression was not detected in the tumours from CXCL14-YCU-H891 cells. However, on day 8, *CXCL14* expression was detected in the tumours formed by the CXCL14-YCU-H891 cells (Figure 4f), indicating that the ectopic expression of CXCL14 itself affected the growth of the tumours derived from YCU-H891 cells.

A schematic representation of the effects of EGF and cetuximab on cell proliferation, and the expression of *CXCL14* is presented in Figure 4g. Even in cancer cells that express *CXCL14*, the expression level is markedly reduced because of EGF/EGFR-binding signals. Here we demonstrated that although cetuximab suppressed the proliferative ability of HSC-3 cells *in vivo*, it did not affect that of YCU-H891 cells. As shown in Figure 2, although YCU-H891 cells contained no mutations in EGFR downstream signalling molecules, the *CXCL14* gene was silenced because of DNA hypermethylation of its promoter (Figure 3). This finding explains why the administration of cetuximab did not lead to a recovery of *CXCL14* expression in YCU-H891 cells (cf. Figure 4g). The present study has demonstrated that cetuximab exhibited tumour-suppressive effects when administered with DAC to mice-bearing YCU-H891 tumours. Thus, hypermethylation of the *CXCL14* promoter may represent a promising biomarker to aid in treatment decisions concerning whether cetuximab and DAC should be administered concurrently. The co-administration of cetuximab and DAC inhibited tumour growth to a greater extent than the administration of DAC alone, suggesting that DAC demethylated the promoter of the *CXCL14* gene, reactivating the gene and that cetuximab, an EGFR inhibitor, further inhibited ERK/MAP kinase signalling, thus stimulating transcription of *CXCL14* gene, as shown in the Figure 4g. The effect of DAC on tumour suppression may also have depended on the expression of some unknown tumour suppressor in addition to *CXCL14*. However the data in Figure 4e, indicate that the introduction of additional *CXCL14* genes into YCU-H891 cells restored the suppression of tumour growth, in the absence of cetuximab treatment and clearly indicates that *CXCL14* expression has a significant effect on tumour suppression *in vivo*.

On the basis of the results obtained in this study, we suggest that to predict the effectiveness of cetuximab before its administration, one must consider not only genetic modifications but also biomarkers such as *CXCL14*, whose expression is silenced by DNA hypermethylation in certain cancer cells.

Here we demonstrated that cetuximab-dependent tumour-suppressive effects in HNSCC cells were dependent on the gene expression of *CXCL14*. Colorectal carcinogenesis is also suppressed in transgenic mice expressing higher levels of the CXCL14 molecule,^44^ as well as in tumour metastasis, suggesting that *CXCL14* expression may represent a biomarker of adenocarcinomas, such as colorectal cancer, although this possibility requires further investigation.

In conclusion, we demonstrated that certain cancer cells do not respond to cetuximab, even in the absence of genetic mutations that prevent the effects of cetuximab, and that cetuximab resistance in these cancer cells may be attributable to DNA hypermethylation of the *CXCL14* gene. Accordingly, it is important to investigate DNA hypermethylation of *CXCL14* promoter regions in patients with HNSCC before cetuximab administration. If the promoter regions of *CXCL14* are methylated, DAC may be used concurrently with cetuximab. In the future, concurrent cetuximab and DAC therapy may represent a novel therapeutic approach for the treatment of malignant tumours that exhibit cetuximab resistance.

## Animal studies

All of the animal experiments were performed in accordance with the protocols approved by the Institutional Animal Care and Use Committee of Kanagawa Dental University and followed the guidelines for animal research issued by the International Association for the Study of Pain Committee for Research and Ethical Issues.

## Human subjects

The cells were obtained in accordance with the protocols approved by Ethics Review Board of Yokohama City University, Medical School.
